# Malaria and urbanization in sub-Saharan Africa

**DOI:** 10.1186/1475-2875-4-12

**Published:** 2005-02-18

**Authors:** Martin J Donnelly, PJ McCall, Christian Lengeler, Imelda Bates, Umberto D'Alessandro, Guy Barnish, Flemming Konradsen, Eveline Klinkenberg, Harold Townson, Jean-Francois Trape, Ian M Hastings, Clifford Mutero

**Affiliations:** 1 Liverpool School of Tropical Medicine, Pembroke Place, Liverpool, L3 5QA. UK; 2 Swiss Tropical Institute, P.O. Box, 4002 Basel, Switzerland; 3 Prince Leopold Institute of Tropical Medicine, Nationalestraat 155, B-2000 Antwerp, Belgium; 4 International Water Management Institute Sri Lanka127, Sunil Mawatha, Pelawatte, Battaramulla, Sri Lanka; 5 Institute of Public Health, Department of International Health, University of Copenhagen, Blegdamsvej 32200 København, Denmark; 6 International Water Management Institute (West Africa), PMB CT 112, Cantonments, Accra, Ghana; 7 Institut de Recherche pour le Développement, BP 1386, CP 18524, Dakar, Sénégal; 8 Systemwide Initiative on Malaria and Agriculture, International Water Management Institute, Private Bag X813, Silverton 0127, South Africa

## Abstract

There are already 40 cities in Africa with over 1 million inhabitants and the United Nations Environmental Programme estimates that by 2025 over 800 million people will live in urban areas. Recognizing that malaria control can improve the health of the vulnerable and remove a major obstacle to their economic development, the Malaria Knowledge Programme of the Liverpool School of Tropical Medicine and the Systemwide Initiative on Malaria and Agriculture convened a multi-sectoral technical consultation on urban malaria in Pretoria, South Africa from 2nd to 4th December, 2004. The aim of the meeting was to identify strategies for the assessment and control of urban malaria. This commentary reflects the discussions held during the meeting and aims to inform researchers and policy makers of the potential for containing and reversing the emerging problem of urban malaria.

## Introduction

Africa's population will almost triple by the year 2050. This expansion will occur primarily in urban areas and by 2025, 800 million people will live in urban communities. Especially affected will be West Africa, where the urban population annual growth rate of 6.3% is more than twice the rate of the total population growth. Today in the humid forest zone, more people live in cities than in rural areas and in twenty years time, two out of three West Africans will live in urban centres. While many of Africa's health problems are common to both urban and rural environments, recognizing and meeting the public health challenges in these growing cities is becoming increasingly urgent. Malaria has been considered a predominantly rural disease in Africa, primarily because suitable vector breeding sites are scarce in highly populated areas. Yet, although studies have shown that *Anopheles *mosquito breeding decreases with increasing proximity to the centre of urban areas [1-3], transmission of malaria still occurs. Clearly, the complex factors contributing to malaria risk in urban areas are not fully understood [3] but evidence is rapidly accumulating that the urban poor are at far higher risk from malaria than previously acknowledged [4,5].

The Malaria Knowledge Programme of the Liverpool School of Tropical Medicine and the International Water Management Institute/ Systemwide Initiative on Malaria and Agriculture convened a meeting in Pretoria 2^nd^-4^th ^December 2004 to develop an evidence-based approach for evaluating and controlling urban malaria. Participants were drawn from seven sub-Saharan countries, Europe, North America and South Asia (see additional file). Recognizing the need for extensive cross-sectoral involvement and collaboration in dealing with the challenge of urban malaria, representatives from the research/ academic, NGO, development, policy-making and donor communities co-operated in the process to identify key knowledge gaps and opportunities for control. Included in the group were sociologists, clinical epidemiologists, entomologists and control specialists.

## Discussion

### Identifying the populations at risk in urban areas

Urbanization is a recent phenomenon in Africa: in 1960 there were no African cities with one million inhabitants, today there are forty. Has malaria become a serious problem within these huge cities and their peri-urban environs? Data presented from studies in a number of sub-Saharan African cities (Brazzavile, Congo; Dakar, Senegal; Abidjan, Cote d'Ivoire; Cotonou, Benin; Ouagadougou, Burkina Faso; Dar es Salaam, Tanzania, and Accra and Kumasi, Ghana) showed clearly that malaria is a considerable urban health problem in Africa. The studies demonstrated great heterogeneities in malariometric indices both between and within cities. It was recognized that not only the major cities of Africa, but also many medium sized regional towns, home to a large proportion of the Africa population, have considerable levels of malaria [5]. With malaria risk unevenly distributed across urban environments, interventions must be preceded by the identification and prioritization of the most vulnerable. Vulnerability is not simply the result of low socio-economic status [6], although this is often a major contributory factor, but reflects factors beyond the individual level such as the proximity of the household to sites of urban agriculture or environmental/cultural factors working at the community level. Discussion focussed on research to define this risk, to improve access to correct diagnosis and appropriate treatment and effective preventative measures, and to identify accurate monitoring and evaluation tools tailored to the urban context.

### Prioritizing improved diagnosis and treatment for the vulnerable

Misdiagnosis of malaria is a serious problem everywhere, but in areas of low malaria endemicity presumptive treatment of all fevers as malaria can result in over 75% of cases being misdiagnosed as malaria [7]. The effect of malaria misdiagnosis on the vulnerable will result in more ill health due to delayed diagnosis and repeat visits, overburdened health services, more severe malaria, loss of faith in health services, increase in real and perceived malaria resistance, chronic disease secondary to untreated infection, increased cost to patient and to health facilities and consistent misdiagnosis that will encourage detrimental health-seeking behaviour [7].

Effective provision of appropriate treatment also remains a serious challenge in urban settings. The Abuja Declaration stated that by 2005 "At least 60% of those suffering from malaria have prompt access to and are able to use correct, affordable and appropriate treatment within 24 hours of onset of symptoms." Despite the fact that access to quality health care is better on average in urban compared to rural zones, the formal public health facilities are often the last source of treatment used along the pathway to cure. Often malaria care initially involves leftover medicines from the home (from previously incomplete malaria or other treatment regimes), the purchase of cheaper herbal medicines or unprescribed conventional medicines. The problems of obtaining treatment from a health facility may be exacerbated by the need to obtain permission from an authority figure, absence from work and loss of income, the need to raise money to fund both the treatment and associated costs such as travel [6]. As a result, in Africa over 70% of malaria episodes in rural and over 50% in urban areas are self-diagnosed and self-treated [8]. With Home Management of Malaria proposed as an integral part of the Roll Back Malaria strategy, the consequences of presumptive treatment policies for malaria in the context of the introduction of newer and more expensive anti-malarial drug combinations urgently require further investigation [9].

### Ensuring malaria prevention measures reach the vulnerable

The highly focal nature of urban malaria requires targeting of interventions to specific urban districts, and therefore, requires detailed information on each area in advance. However, relationships between administrative boundaries, environment and population distribution are complex in urban areas, which makes them difficult to sample and characterize in a representative way. Strategies for population-representative sampling must incorporate a range of environments and populations to identify accurately environmental and other risk factors. This may be further complicated as urban populations can be highly mobile and in peri-urban areas there may be a high rate of turnover in groups of lower socio-economic status. Presentations from South, East and West Africa clearly demonstrated that Geographic Information Systems (GIS)-based approaches are valuable tools for assessing heterogeneities in risk factors for urban malaria, and for subsequent implementation and monitoring of interventions.

Experienced researchers believe that the urban environment has advantages for the effective delivery of appropriate interventions. A number of studies have demonstrated that higher rates of coverage with insecticide-treated bednets can be achieved in urban areas [10,11], although whether or not the most vulnerable groups benefit, remains to be confirmed. Moreover, there is a growing realization within the commercial sector of the need to engage in health and broader social issues. The management of malaria can bring economic benefits to both businesses and the communities in which they operate. This has been powerfully demonstrated in two public-private partnership programmes in southern Africa that utilised indoor residual spraying to control malaria [12,13].

Larval control, achieved either by source reduction or larviciding, can be community directed and may be feasible in certain settings as part of a comprehensive, integrated vector management strategy. There is optimism in some communities about its efficacy and the results of further research into the costs and benefits of such interventions are awaited with interest. Environmental modifications may also be feasible if partners from the community and outside the health sector are engaged. Work from Sri Lanka has demonstrated how a very effective scheme to control malaria by modification of irrigation structures was accepted by the agricultural community because of the financial and water savings that the scheme introduced [14]. However, it was clear that obvious benefits from the intervention must exist to attract the involvement of non-health sectors.

## Conclusions

**Figure 1:**
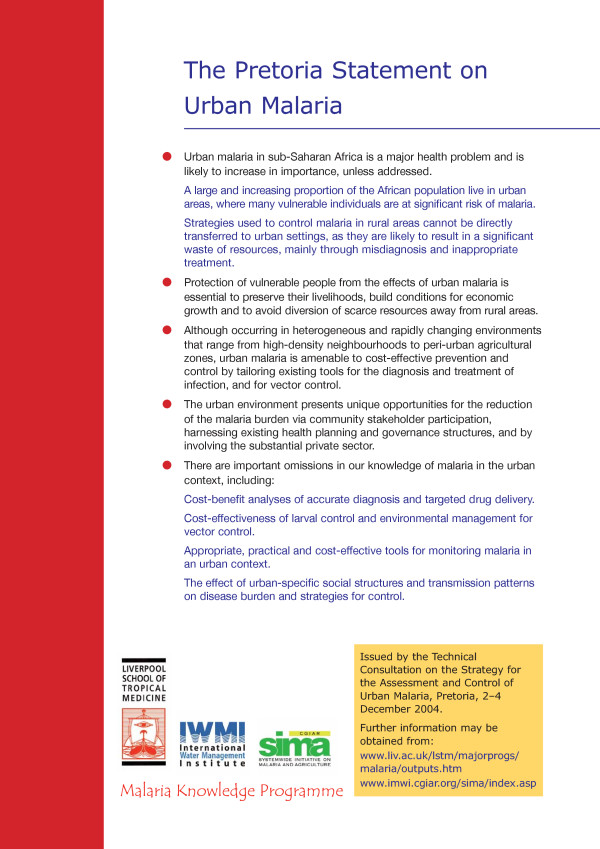
The Pretoria Statement on Urban Malaria.

The conclusions of the meeting have been summarised in the Pretoria Statement on Urban Malaria (Figure 1). While it is clear that urban malaria represents a major challenge for public health in Africa, the statement highlights that the unique nature of the urban environment provides an opportunity for malaria control. There are a number of reasons for this: the high population density in urban areas may facilitate increased coverage and impact of both interventions and health education programmes; the activities of departments in urban municipal authorities are typically better resourced and more easily mobilized than in rural areas; the extensive private health sector found in urban settings can be engaged to improve diagnosis, treatment and prevention of malaria. Solutions to the urban malaria problem must include groups from outside the health sector. The disease burden in the most vulnerable communities is a major obstacle to the economic growth of sub-Saharan countries and the challenge is to engage stakeholders at all levels in effective and sustainable intersectoral collaboration [15]. Urban malaria is uniquely amenable to prevention and control as the existing health, urban planning, agricultural and governance structures present opportunities for collaborative approaches that can include both the community and the substantial private sector.

## Authors' contributions

All authors participated as session chairs in the technical workshop and were instrumental in producing the summary conclusions. All authors read and approved the final manuscript.

## Supplementary Material

Additional File 1 List of attendees and affiliations Click here for file
