# Supplementary material for: Malaria and urbanization in sub-Saharan Africa
Source: Malar J. 2005 Feb 18;4:12. doi: 10.1186/1475-2875-4-12 (PMC552321; doi:10.1186/1475-2875-4-12)
Supplement: Additional File 1 — List of attendees and affiliations [file 1475-2875-4-12-S1.doc]

Appendix 1 List of attendees and affiliations

| **Name** | **Affiliation** | **Contact details** |
| --- | --- | --- |
| Umberto d’Alessandro | Prince Leopold Institute of Tropical Medicine, Nationalestraat 155m B-2000 Antwerp BELGIUM. | Tel: +32 3 247 63 54  Fax: +32 3 247 63 59  Email: [udalessandro@itg.be](mailto:udalessandro@itg.be) |
| Guy Barnish | Liverpool School of Tropical Medicine, Pembroke Place, Liverpool, L3 5QA.  UNITED KINGDOM | Tel: +44 151 705 3153  Fax: +44 151 705 3369  Email: [gbarnish@liv.ac.uk](mailto:gbarnish@liv.ac.uk) |
| Imelda Bates | Liverpool School of Tropical Medicine, Pembroke Place, Liverpool, L3 5QA.  UNITED KINGDOM | Tel: +44 151 705 3115  Fax: +44 151 705 3369  Email: [ibates@liverpool.ac.uk](mailto:ibates@liverpool.ac.uk) |
| Gene Brantly | International Development Group  RTI International, Inc.  1615 M Street N.W., Suite 740  Washington, D.C. 20036-3209  UNITED STATES | Tel: +1 202-974-7801 (direct phone)  Tel: +1 202-728-2080 (attended line)  Fax: +202-728-2475  Email: [epb@rti.org](mailto:epb@rti.org) |
| Charles Delacollette (could not attend) | Roll Back Malaria  HTM/WHO  Geneva  SWITZERLAND | Tel: +4122 791 2766  Fax:+4122 791 4824  Email: [delacollettec@who.int](mailto:delacollettec@who.int) |
| Stefan Dongus | University of Freiburg  Department of Physical Geography  Section on Applied Geography of the Tropics and Subtropics (APT)  Werderring 4  79085 Freiburg, GERMANY | Tel: +49-761-2039126  Fax: +49-761-2033508  Email: [stefan.dongus@geographie.uni-freiburg.de](mailto:stefan.dongus@geographie.uni-freiburg.de) |
| Martin Donnelly | Liverpool School of Tropical Medicine, Pembroke Place, Liverpool, L3 5QA.  UNITED KINGDOM | Tel: +44 151 705 3296  Fax: +44 151 705 3369  Email: [mjames@liv.ac.uk](mailto:mjames@liv.ac.uk) |
| Alison Dunn | Exchange / Healthlink Worldwide  56-64 Leonard Street  London  EC2A 4JX  UNITED KINGDOM | Tel: +44 207 549 0250  Fax: +44 207 549 0241  Email: [dunn.a@healthlink.org.uk](mailto:dunn.a@healthlink.org.uk) |
| Ulrike Fillinger | University of Durham, School of Biological and Biomedical Sciences, Durham  UNITED KINGDOM | Email: [ulrike.fillinger@durham.ac.uk](mailto:ulrike.fillinger@durham.ac.uk) |
| John Githure  (could attend) | International Centre of Insect Physiology and Ecology, P.O. Box 30772, Nairobi, KENYA | Tel: +254 (20) 802-501/861680  Fax: +254 (20) 860-110  Email: [jgithure@icipe.org](mailto:jgithure@icipe.org) |
| Ian Hastings | Liverpool School of Tropical Medicine, Pembroke Place, Liverpool, L3 5QA.  UNITED KINGDOM | Tel: +44 151 705 3183  Fax: +44 151 705 3369  Email: [hastings@liv.ac.uk](mailto:hastings@liv.ac.uk) |
| Lucy Kabuage | Department of Animal Production  University of Nairobi  P.O. Box 29053  Nairobi, KENYA | Tel: +254-2-631340/631277  Fax: +254-2-631487  Email: [kabuage@gt.co.ke](mailto:kabuage@gt.co.ke) |
| Charity Kabutha | Human Health Division  International Centre of Insect Physiology and Ecology (ICIPE)  P.O. Box 30772  Nairobi, KENYA | Tel: +254 722562638  Email: [c_kabutha@yahoo.com](mailto:c_kabutha@yahoo.com) |
| Khadija Kannady | PO Box 22175  Dar Es Salaam  TANZANIA | Email: [kadkhadija@yahoo.co.uk](mailto:kadkhadija@yahoo.co.uk) |
| Gerry Killeen | Ifakara Health Research and Development Centre  PO Box 53  Ifakara, Kilombero District  TANZANIA | Tel:+255-23-262-5164  Fax:+255-23-262-5312  E-mail: Gerry.Killeen@unibas.ch |
| Eveline Klinkenberg | International Water Management Institute (IWMI)  PMB CT 112, Cantonments, Accra  GHANA    After 02/05  Liverpool School of Tropical Medicine, Pembroke Place, Liverpool, L3 5QA  UNITED KINGDOM | Tel: +233 21 784753  Fax: +233 21 784752  Email: [e.klinkenberg@cgiar.org](mailto:e.klinkenberg@cgiar.org)  Email:evelinek@liv.ac.uk |
| Flemming Konradsen | Department of international Health  Institute of Public Health  University of Copenhagen  Blegdamsvej 3  DK-2200 Copenhagen N  DENMARK  Based at:  IWMI, P.O. Box 2075, Colombo , SRI LANKA | Tel: +45-35327776  Fax: +45-35327736  Email: [f.konradsen@pubhealth.ku.dk](mailto:f.konradsen@pubhealth.ku.dk)  Email: [f.konradsen@cgiar.org](mailto:f.konradsen@cgiar.org) |
| Christian Lengeler | Swiss Tropical Institute, P.O. Box, 4002 Basel, SWITZERLAND | Tel: +41 61 284 8221  Email: [Christian.Lengeler@unibas.ch](mailto:Christian.Lengeler@unibas.ch) |
| Matt Lynch | Malaria Advisor USAID  Office of Health, Infectious Diseases & Nutrition Bureau for Global Health  Washington, DC  20523-3700  UNITED STATES | Tel: +1 202 712 0644  Email: [MLynch@usaid.gov](mailto:MLynch@usaid.gov) |
| Rajendra Maharaja | Malaria Research Programme,  Medical Research Council  491 Ridge Road  Overport, 4067, SOUTH AFRICA | Tel: +27 31 203 4803  Fax: +21 31 203 4704  Email: [raj.maharaj@mrc.ac.za](mailto:raj.maharaj@mrc.ac.za) |
| Evan Mathenge | PO Box 16619  00100GPO  Nairobi, KENYA | Email: [mathengeevan@yahoo.co.uk](mailto:mathengeevan@yahoo.co.uk) |
| Charles Mbogo | KEMRI Wellcome Trust Research Programme  Off Mombasa-Malindi Road  PO Box 428  80108 Kilifi, KENYA | Fax: +254 41522390  Email: [cmbogo@kilifi.minicom.ke](mailto:cmbogo@kilifi.minicom.ke) |
| Philip McCall | Liverpool School of Tropical Medicine, Pembroke Place, Liverpool, L3 5QA,  UNITED KINGDOM | Tel: +44 151 705 3132  Fax: +44 151 705 3369  Email:mccall@liv.ac.uk |
| Maureen Mubanga | CARE International-Zambia  P.O. Box 36238 Plot No. 10799/10800  Dedan Kimathi Road, Kamwala  Lusaka, ZAMBIA | Tel: 260 1 221687  Fax: +260 1 222564/222563  Email: [mubanga@carezam.org](mailto:mubanga@carezam.org) |
| Clifford Mutero | Systemwide Initiative on Malaria and Agriculture c/o International Water Management Institute, Private Bag X813, Silverton 0127, SOUTH AFRICA | Tel: +2712 845 9100  Fax: +2712 845 9110  Email: [c.mutero@cgiar.org](mailto:c.mutero@cgiar.org) |
| Tendani Nevondo | Systemwide Initiative on Malaria and Agriculture c/o International Water Management Institute, Private Bag X813, Silverton 0127, SOUTH AFRICA | Tel: +27 12 845 9100  Fax:+27 12 845 9110  Email: [t.nevondo@cgiar.org](mailto:t.nevondo@cgiar.org) |
| Joseph Okello-Onen | Senior Research Officer  (Veterinary Entomologist)  Livestock Health Research Institute (ILRI), P.O. Box 96  Tororo, UGANDA | Tel: +256 77 353223  Fax: +256 45 45052  [Email: jonen65@hotmail.com](mailto:jonen65@hotmail.com) |
| Renaud de Plaen | Écosystèmes et Santé Humaine/Ecosystem Approaches to Human Health, CRDI/IDRC  250 Albert street  Po. Box 8500, Ottawa, CANADA, K1G 3H9 | Tel: +1 (613) 236-6163 ext. 2545  Fax: 1 (613) 567-7748  E-mail: [rdeplaen@idrc.ca](mailto:rdeplaen@idrc.ca) |
| Brian Sharp | Malaria Research Programme  Medical Research Council  491 Ridge Road  Overport, 4067  SOUTH AFRICA | Tel: +27 31 024 3600  Email: [sharpb@mrc.ac.za](mailto:sharpb@mrc.ac.za) |
| Jose Siri | University of Michigan  632 Church St.  Ann Arbor, MI 48104 | Tel: +1 734-657-8329  E-mail: [jsiri@umich.edu](mailto:jsiri@umich.edu) |
| Seter Siziya | University of Zambia  School of Medicine  Department of Community Medicine, P O Box 50110  Lusaka, ZAMBIA | Phone: +260-96-748988  TelFax: +260-1-256181  E-mail: [ssiziya@yahoo.com](mailto:ssiziya@yahoo.com) |
| Stella Talisuna | Uganda Virus Research Institute,  Nakiwogo Road, Plot 51-59,  P.O.BOX. 49 Entebbe, Uganda, | Tel: +256 77 22 88 44.  Fax: +256 41 320483.  Email: [mrstalisuna@yahoo.com](mailto:mrstalisuna@yahoo.com) |
| Harold Townson | Liverpool School of Tropical Medicine, Pembroke Place, Liverpool, L3 5QA | Tel: +151 705 3124  Fax: +151 705 3369  Email: [htownson@liv.ac.uk](mailto:htownson@liv.ac.uk) |
| Jean-Francois Trape | IRD, BP 1386,  CP 18524  Dakar, Senegal | Tel: +221 849 35 82 / 84  Fax: +221 832 43 07  Email: [Jean-Francois.Trape@ird.sn](mailto:Jean-Francois.Trape@ird.sn) |
| Brishore K Tyagi | Centre for Research in Medical Entomology (ICMR)  4,Sarojini Street, Chinna Chokkikulam,  MADURAI 625002 (TN), India | Tel: +91-0452-2525668  -2644247  Fax:+91-0452-2530660  Email: [divccrme@sify.com](mailto:divccrme@sify.com) |
| Henk van den Berg | UNEP Chemicals  International House of Environment  Geneva, Switzerland    Based at:  Entomology Department  Wageningen University & Researchcentre  P.O. Box 8031  6700 EH Wageningen  The Netherlands | Tel: +31 317 482328 or 452302  Email: [Henk.vandenBerg@wur.nl](mailto:Henk.vandenBerg@wur.nl) |
| Daniel Gatheru Wacira  (could not attend) | African Medical and Research Foundation  P.O. Box 30125 Nairobi, Kenya | Tel: +254 604651-9  Fax: Fax 254-20-606340  Email: [dgatheru@hotmail.com](mailto:dgatheru@hotmail.com) |
| Michael Wilson | Noguchi Memorial Institute for Medical Resarch  University of Ghana PO Box 25, Legon, Ghana | Email: [MWilson@noguchi.mimcom.net](mailto:MWilson@noguchi.mimcom.net) |
